# Variability of Micro- and Macro-Elements in Anaerobic Co-Digestion of Municipal Sewage Sludge and Food Industrial By-Products

**DOI:** 10.3390/ijerph20075405

**Published:** 2023-04-05

**Authors:** Aleksandra Szaja, Agnieszka Montusiewicz, Magdalena Lebiocka

**Affiliations:** Faculty of Environmental Engineering, Lublin University of Technology, Nadbystrzycka 40B, 20-618 Lublin, Poland

**Keywords:** anaerobic co-digestion, heavy metals, nutrients content, orange juice wastes, brewery spent grain

## Abstract

The main aim of this study was to evaluate the effect of the addition of selected industrial food wastes on the fate of micro- and macro-elements within an anaerobic digestion process (AD), as well as define the relationship between their content and AD efficiency. Orange peels, (OP), orange pulp (PL) and brewery spent grain (BSG) were used as co-substrates, while municipal sewage sludge (SS) was applied as the main component. The introduction of co-substrates resulted in improvements in feedstock composition in terms of macro-elements, with a simultaneous decrease in the content of HMs (heavy metals). Such beneficial effects led to enhanced methane production, and improved process performance at the highest doses of PL and BSG. In turn, reduced biogas and methane production was found in the three-component digestion mixtures in the presence of OP and BSG; therein, the highest accumulation of most HMs within the process was also revealed. Considering the agricultural application of all digestates, exceedances for Cu, Zn and Hg were recorded, thereby excluding their further use for that purpose.

## 1. Introduction

In recent years, anaerobic co-digestion (AcoD) has become an important method for renewable energy generation, effective waste management, as well as the production of nutrient-rich digestate. It allows overcoming the difficulties of mono-digestion resulting from an imbalanced C/N ratio, a lack of micro- and macro-nutrients, the presence of inhibitors, and low organic loads [1]. There are many benefits to be gained from using this strategy, e.g., enhanced biogas production, improved digestate quality and more stable process performance [2]. Despite many successful implementations at existing wastewater treatment and biogas plants, there are still many difficulties that are related mainly to process stability. The microorganisms involved in anaerobic digestion (AD) vary considerably in terms of nutritional needs, growth kinetics, physiology and sensitivity to environmental conditions [3]. Both heavy metals and light metal ions are considered to be the strongest process inhibitors; however, their harmfulness is determined by their concentration and form. On the other hand, at adequate concentrations, these substances may have a favorable influence on the AD microorganisms [4,5].

Another relevant aspect is the influence of the substrates used on the quality of the digestate. These by-products are rich in essential elements and nutrients, such as nitrogen (N), phosphorus (P), potassium (K), amino acids and vitamins, that may improve soil fertility [6]. However, their agricultural application is limited mainly by the presence of HMs, and the potential transmission of HMs to human beings [7,8,9]. With this in mind, the successful application of AcoD requires the analysis of many aspects, not only biogas production, but also possible environmental risks resulting from the properties of the applied substrates.

Among the various wastes, by-products of the food and drink processing industry constitute a source of many potential substrates that can be applied in the AD process. It is estimated that in the EU, this is the largest industrial sector, with a turnover of EUR 1.1 trillion [10]. Moreover, this sector is responsible for the generation of significant amounts of residues, the effective management of which is still a challenge. Among this group, the residues from orange juice and beer production constitute a significant share. The first is represented mostly by orange peel (OP) and pulp (PL), whereas the second is represented by brewery spent grain (BSG). The first two have been used as fertilizer, animal feed, charcoal production, as well as for the extraction of essential oils and pectin [11]. In turn, BSG has been applied as livestock feed, or as raw material for the production of enzymes, biopolymers, biocides and biofuel [12].

Taking into consideration the significant energy consumption of the food industry, the application of such wastes in AD processes becomes a favorable choice. However, the anaerobic transformation of these wastes is still a technological problem [13,14]. They may contain substances that are recognized as AD inhibitors, e.g., terpenes, phenols and ammonium [3]. Additionally, some wastes are lignocellulosic in nature, which indicates poor degradability within anaerobic transformation [14]. On the other hand, both substrates are rich in many vitamins, such as C, A and B, minerals, amino acids and supply a high organic load that may stimulate methanogenic activity. Moreover, their application may improve digestate quality [15,16,17].

The main aims of this study were the following: (1) to evaluate the effect of the addition of selected industrial food wastes on the fate of micro- and macro-elements within anaerobic transformation; (2) to examine the effect of co-substrate application on biogas/methane production, volatile solids removal (VRS) as well as process stability; (3) to define the relationship with HMs content and biogas production; (4) to determine the possibility of the digestate for agricultural use.

In the present study, the main solid by-products of orange juice and beer processing were chosen as the food industrial wastes. These residues, represented by OP, PL and BSG, were selected taking into consideration their significant amounts, and the ongoing problem with their effective management. Moreover, all of these substances indicated a potential for biogas production [13]. The co-substrates were added to municipal sewage sludge (SS), the main by-product of wastewater processing. The experiment was conducted in batch reactors under mesophilic conditions. The multi-component mixtures were examined. It should be noted that in terms of these substrates, similar investigations have not been conducted previously. The basic novelty of this study is that it presents a holistic approach, in which many factors have been taken into account, i.e., biogas production, process efficiency as well as environmental aspects of the proposed technology. Importantly, the obtained results may be implemented on a technical scale, thus improving the energy balance in many wastewater treatment plants (WWTPs). Moreover, for food producers, the application of this technology may allow for effective waste management that is consistent with the assumptions of the circular economy.

## 2. Materials and Methods

### 2.1. Preparation of Substrates

The inoculum and SS used in the present study originated from a mechanical–biological municipal WWTP located in Lublin (Poland), with an average daily flow of 65,000 m^3^/d. After collection, these were transported to the laboratory, and then screened to remove major particles. At first, the inoculum was added to batch reactors in the amount of 1.4 dm^3^, flushed with nitrogen to provide anaerobic conditions, and maintained at a temperature of 37 °C to reduce non-specific biogas production within the experiment. After this, the substrates were added to reactors.

The SS was obtained by mixing thickened primary and excess sludge in a volumetric ratio of 60:40 *v/v* and characterized by total solids (TS) of 44.8 ± 3.9 g/kg, volatile solids (VS) of 33.85 ± 2.9 g/kg and a pH of 6.43 ± 0.1. The adopted proportion is typical for Polish conditions, and it allowed for effective biogas production. Inoculum was taken from the WWTP mesophilic digester effluent and characterized by the following parameters: TS of 18.85 ± 0.23 g/kg, VS of 9.95 ± 0.16 g/kg and a pH of 7.9 ± 0.1.

The by-products from orange juice and beer production were applied as industrial food wastes. These wastes were selected, bearing in mind the ongoing problem with their effective management and their potential for biogas production.

The oranges for the experiment were taken from a local market. In turn, BSG was taken from a craft brewery located in Lublin, Poland. The applied co-substrates were pre-treated only to the extent that they could supply the batch reactors of a small volume of 2 L.

Before squeezing the juice, the oranges were washed as in the case of industrial production. Before supplying the reactors, the OPs were cut to achieve a particle size of 0.5 cm. The second type of orange waste was represented by PL; this sample was obtained as a by-product of the laboratory juice extractor, and mainly consisted of fibers and fruit flesh.

BSG was a solid fraction obtained during wort generation in the brewing manufacturing process. Before supplying the batch reactors, this sample was also crushed to a particle size of 2–5 mm. The detailed compositions of the co-substrates used in experiment are presented in Table 1.

### 2.2. BMP Assays

**Figure 1:**
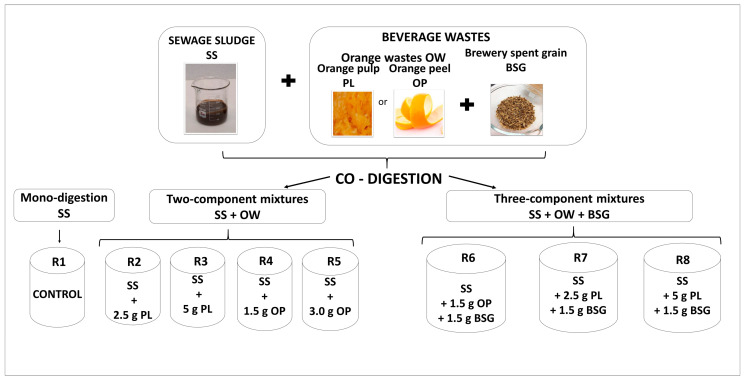
The experimental set-up applied in the present study.

The BMP assays were carried out in batch reactors with an active volume of 2.0 L, provided by BPC Instruments AB (Sweden). The experiment was conducted under mesophilic conditions at a temperature of 37 °C. The 8 batch reactors (R1-R8) applied in this study differed in their feedstock compositions. Each vessel was supplied by 1.4 L of inoculum and 0.4 L of SS. In this study, I:S (inoculum to substrate ratio) was established at a level of 1.7. In the co-digestion series, the following doses were evaluated: 1.5 and 3.0 g of OP, 2.5 and 5 g of PL and 1.5 g of BSG. In turn, to compare the obtained results, a control reactor was provided, where mono-digestion was conducted (R1). The adopted doses were established on the basis of the VS of the substrates. It should be pointed out that both OP and BSG indicated a relatively high content of VS as compared to SS. Additionally, the application of food wastes that contain significant amounts of organic matter may lead to digester overloading, resulting in digester acidification [1]. Figure 1 illustrates the detailed operational set-up applied in the present study.

### 2.3. Analytical Methods

The samples of feedstock and digestate were taken to determine their chemical and physical properties, and the following parameters were examined: chemical oxygen demand (COD), TS, VS, pH, volatile fatty acid (VFA) and total alkalinity (ALK). Moreover, the contents of macro- (N and P, K, Mg, P, K, Ca and S) and micro-elements (Cr, Mn, Fe, Ni, Cu, Zn, As, Cd, Hg and Pb) were analyzed.

The TS and VS contents were determined based on the procedure presented in the Standard Methods for the Examination of Water and Wastewater [18]. Other analyses were conducted involving a Hach Lange UV–VIS DR 5000 spectrophotometer (Loveland, CO, USA) and standard cuvette tests. The pH values were controlled by a CPC 501 multimeter (Elmetron, Poland). To analyze the VFA and ALK contents, the samples were centrifuged at 4000 r/min for 30 min; the obtained supernatant was passed through a microfiber filter of 0.45 µm.

The methane content in biogas was evaluated using by a ThermoTrace GC-Ultra gas chromatograph (Thermo Fisher Scientific, Milan, Italy) equipped with a conductivity detector fitted with divinylbenzene (DVB)-packed columns (RTQ-Bond). Helium with a flow rate of 1.5 cm^3^/min was used as a carrier gas, and the applied temperatures were 50 °C for the injector and 100 °C for the detector.

An Agilent 8900 ICP-MS Triple Quad inductively coupled plasma mass spectrometer (Agilent, Santa Clara, CA, USA) was used for an elemental analysis. The major analyses were performed in the He mode with a flow rate of 5.5 mL/min. The exceptions were the analyses of Se and As; these were carried out in the O_2_ mode. Before the analyses, wet mineralization of the samples was conducted. In the case of the feedstock and digestate, each 0.1 g of lyophilized sample was mineralized via an addition of 5 mL of 69% Super Purity Acid HNO_3_ (ROMIL), and 2 mL of HCl Suprapur (Merck, Rahway, NJ, USA), followed by heating to 190 °C in closed Teflon containers of the TOPEX microwave mineralization system (PreeKem, Shanghai, China). Finally, these samples were diluted to 25 mL by ultrapure water obtained in the Milli-Q purification system (Millipore, Darmstadt Germany). The co-substrate wet mineralization of each 0.5 g sample was performed via the addition of 7 mL of 69% Super Purity Acid HNO_3_ (ROMIL), followed by heating to 190 °C in closed Teflon containers of the TOPEX microwave mineralization system (PreeKem, Shanghai, China). After mineralization, 1 mL of HCl Suprapur (Merck) was added to stabilize some of the elements, e.g., As, Hg. Finally, the samples were diluted to 25 mL by ultrapure water obtained from the Milli-Q purification system (Millipore, Darmstadt, Germany).

The statistical analyses were performed using ANOVA (Statistica v.13); the probability level of *p* < 0.05 was considered as significant. Each experimental series was repeated three times under unchanged operational conditions; the average data are presented in tables and figures.

## 3. Results and Discussion

### 3.1. Digestion Efficiency

As shown in Table 2, the application of co-substrates resulted in improvement in VSR (volatile solids removal), as compared to that for the control reactor. The highest enhancement of approx. 25% was observed at the lowest doses of both orange wastes.

The optimum pH range for AD microorganisms should vary between 6.5 and 8.0. In turn, a VFA/ALK ratio of 0.23–0.3 constitutes a recommended range for stable AD performance [2,19,20,21]. In all of the experimental series, the stability parameters were at a favorable level for the AD process (Table 2). However, in the presence of the highest doses of both orange wastes, the adverse effect on the VFA/ALK ratio was noted. This observation was related with the application of substrates that indicated low ALK and acidic pH (Table 1). It should be noted that in the presence of BSG in three-component digestion, a different trend was achieved. In this case, the introduction of co-substrate with significant ALK provided adequate buffering capacity in the reactors. Such beneficial effects of BSG have been reported previously in the case of co-digestion of acid cheese whey and SS [22].

**Figure 2:**
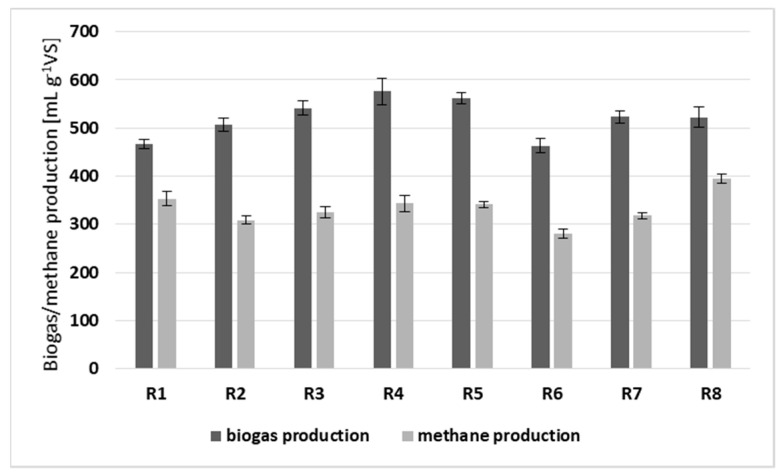
Results in terms of biogas and methane production in the corresponding series (average values and 95% confidence limits are given) [23].

The essential efficiency parameters were the biogas and methane production, as well as the methane content in biogas. As shown in Figure 2, in almost all of the co-digestion series, an improvement in the first and a deterioration in the second were observed, as compared to SS mono-digestion (R1). The significant enhancement in both values related only to the three-component AcoD (R8) supplied by 5 g of PL and 1.5 g BSG. In this case, the average values were 522 and 395 mLg/VS for biogas and methane production, respectively. Beneficial results were also obtained in two-component AcoD in the presence of both OP doses (R4, R5), for which the biogas production was increased by over 21% as compared to the control [23]. Importantly, in this case, the decreased methane content in biogas did not decrease the methane, yielding a value below that for SS mono-digestion. Such a tendency is a common effect in the AcoD of substrates with a significant content of carbohydrates [1]. The decreased methane concentration may be attributed to the limonene presence in OP, which may adversely affect methanogens [24].

**Figure 3:**
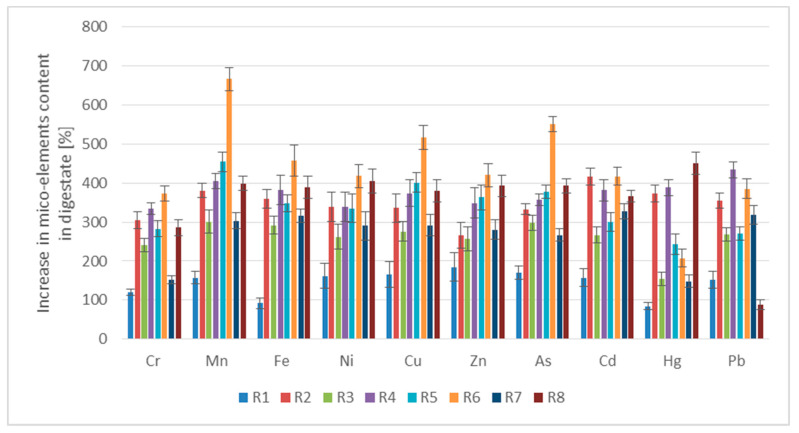
Increase in micro-elements content in digestate within the AD process (average values and standard deviations are given).

It should be pointed out that a decreasing tendency was observed only in the three-component mixtures in the presence of OP (R6). This effect occurred despite a relatively high VRS and favorable VFA/ALK ratio may be related with the presence of both AD inhibitors, such as limonene and phenol compounds, which was confirmed in the authors’ study [23]. Limonene is classified as an essential oil that exhibits toxic effects on AD microorganisms [25]. In turn, acetate-utilizing methanogenesis is particularly sensitive to the inhibitory effects of phenolic compounds [26]. Microbial activity may also be disturbed by high organic loads within the AD of various food wastes [27]. Another question is that in R6, the highest increases in Mn, Fe, Cu, Zn and As were found in digestate (Figure 3), which indicated the relationship between worsening the AcoD efficiency and a deterioration in the digestate quality.

### 3.2. The Variation of Micro- and Macro-Elements within AD

#### 3.2.1. Macro-Elements Content

Balanced feedstock composition is a key factor for effective biogas production. Within the implementation of the AcoD strategy, one of the major decisions involves selecting an adequate co-substrate with a complementary composition to the SS [1,2]. Therefore, conducting analyses of nutrients, metalloids as well as HMs presented in applied materials is particularly important [28].

The characteristics of sole SS may differ remarkably, depending on the wastewater characteristics, as well as the treatment technology adopted at WWTPs. Properly selected co-substrates may significantly change the feedstock composition, in particular, supplying deficient micro- and macro-elements to the main component [2,29].

Among the macro-elements of great importance are considered nitrogen (N), phosphorus (P), potassium (K), sulfur (S), calcium (Ca), magnesium (Mg) and sodium (Na). These components are essential for the microbial growth of microorganisms within AD [30].

The application of the co-substrates resulted in increased contents of macro-elements in the feedstock (Table 3), as compared to the control (R1), in most cases visibly enriching its composition. The exception was the K content in R5, as well as the S content in R2 and R3, both being lower than that for the control. The highest enhancements were observed in the three-component series in the presence of both PL doses and BSG, i.e., for R7 and R8, respectively. This effect was related with the introduction of an additional component in BSG, indicating a high content of TS, VS as well as N, Na, P and S (Table 1). Additionally, the applied PL, as compared to OP, indicated a more valuable composition in terms of TN, P and K contents. Moreover, this orange waste was characterized by higher concentrations of both ALK and COD (Table 1).

All of the analyzed macro-elements play an important role within AD. However, a decisive influence on the AD effectiveness corresponds to N, being a crucial nutrient in protein biosynthesis. Its low concentration may lead to a reduction in the metabolic capacity [31]. On the other hand, a high N content may result in ammonia inhibition [3]. It is commonly known that the preferred C/N ratio in feedstock should be between 20–30:1 [31,32]. In the present research, values above 20 were reached only in the mono- and two-component digestions (R1–R5). In turn, the highest methane production was achieved in the three-component series (R8), despite the unfavorable C/N ratio. Often, the application of co-substrates, and hence supplying the digesters with various micro- and macro-elements, may lead to enhanced process performance (despite disadvantageous conditions, e.g., low pH, adverse VFA/ALK or C/N ratios). In an anaerobic co-digestion strategy, this influence is known as synergistic effects [2]. Moreover, previous studies also indicated that the toxicity of one compound may be eliminated by the presence of some trace elements in an additional co-substrate. Such a phenomenon was observed in the case of light metals, in particular Na, K and Mg [3,33]. In the present study, all of these were significantly enhanced in the R8 feedstock, as compared to the control (R1), which seems to confirm the above-mentioned regularity.

Phosphorus is also needed for appropriate cell growth. Additionally, it plays an important role in the maintenance of an optimal pH during AD [34]. Although significant improvements of over 150% were reached in almost all of the AcoD series, the content of P was still at a relatively low level [3].

In turn, K indicated a stimulatory effect on the AD microorganisms, especially at low concentrations [3,35]. The highest K content was observed in the three-component AcoD in the presence of PL and BSG (R7 and R8). The application of all co-substrates did not lead to the limit value of 400 mg/L being exceeded, thus allowing for effective biogas production.

The highest values of Mg were reported in the presence of PL for both the two- and three-component AcoD. The related enhancements exceeded 50%. Despite this fact, the Mg content was established at an optimal level below 720 mg/L [36,37].

Similarly to the other macro-elements, the Ca concentration was the highest in the ternary mixtures in the presence of PL and BSG. This metal plays an important role in the formation of microbial aggregates [38,39]. Importantly, in all of the AcoD series, the Ca contents were established at levels considered as stimulatory for AD efficiency (100–1035 mg/L) [3,37,40].

The S content was also enhanced for each case as compared to the control, reaching a comparable level with the highest content in the three-component AD. The observed values were below 800 mg/L, i.e., a concentration known to inhibit the AD process [40]. This macro-element has a strong influence on metabolic activity; the sulfate-reducing bacteria compete with methanogens for intermediate product energy sources [41].

In the case of Na, there was no significant influence of PL application observed on its content (R2 and R3), while using OP (R4, R5) as well as OP and BSG increments above 35% occurred (comparing the values expressed in g/kg). As in the case of other macro-elements, its concentration was established at an optimal ratio below 350 mg/L [42].

While analyzing all of the results, a clear tendency was noticed in R6, i.e., in the presence of OP and BSG. In this case, as compared to the other AcoD series, the minor increases in the nutrient content were accompanied by the lowest methane production (Figure 2). This fact may be related with specific BSG absorption properties. Previous studies indicated that the activated carbon produced from this lignocellulosic waste is characterized by better adsorption properties than many commercial products. Moreover, it has a potential to absorb phenolic compounds, as well as some metal anions, e.g., Ni, Fe, Cr, Cd and Pb, from wastewater [43]. However, limonene still appeared to inhibit the process. A surprisingly different effect was obtained using PL and BSG (R7 and R8). This time, the improved feedstock composition resulted in enhanced biogas production, as well as stable process performance. This fact, in particular, indicated different properties of these orange wastes (PL and OP), and emphasized a synergistic effect occurring in the presence of PL and BSG. Moreover, it should be mentioned that in the two-component mixtures, there were no negative effects on biogas production as well as no increased accumulation of metals within the AD process (Figure 3).

The digestate quality is a key factor regarding its further application, particularly in soil fertilization and remediation. Digested biomass contains macro-elements that are easily available for plants, while the bioavailability of P in digested sludge varies between 40 to 80% [44]. Digested SS may also supply some micronutrients, e.g., amino acids or vitamins that are otherwise not added to the soil. Furthermore, its application may improve the structure of the soil. Such a solution is beneficial regarding environmental and economic aspects; it also implements assumptions of the new Circular Economy Action Plan for a cleaner and a more competitive Europe [45]. However, digestate application is strictly regulated because of contamination risks for HMs, pharmaceuticals, as well as pathogens [28,46,47,48].

As it was shown in the study, increases in almost all of the analyzed macro-elements occurred within AcoD, as compared to the feedstock composition (Table 3). The introduction of co-substrates resulted in increased contents of macro-elements in the digestate. A different trend occurred only in the case of N, for which a reduction in its concentration was observed in the three-component series (R6–R8) compared to that in the control (R1). This fact may be related to the effective N utilization for the synthesis of amino acids and proteins within the AD process. However, the N content in all of the series reached a high value, and was at levels typically observed at Polish WWTPs, ranging between 30.5 to 80.50 g/kg [49,50,51]. While comparing digestates from the different AcoD experiments, one exception was noted concerning series R7, with lower contents of Mg, Ca and S versus those achieved for the control.

Other crucial nutrients in the further application of digested SS included P and K. The former is known as a catalyst in biochemical reactions in plants, while the latter plays an important role in the resistance of plants against various stress conditions in the environment [52]. In all of the AcoD series, their contents were enhanced as compared to SS digestate where mono-digestion was conducted. It should be pointed out that the contents of both macro-elements in all of the experimental series were relatively high, as compared to those of other studies. Typically, digested sludge contains 13.3–16.6 g/kg of P and up to 10 g/kg of K [28]. This difference may have resulted from the characteristics of the wastewater that was discharged to the municipal WWTP. In some Polish studies, a high content of P, ranging up to 30.00 g/kg, was also noticed [49].

Sulfur is another essential nutrient that participates in the regulation of growth and development in plants [53,54]. As in the case of the other analyzed nutrients, in all of the AcoD series its content was improved, and reached a relatively high level. At municipal WWTPs, it typically varies between 11 and 13.5 g/kg [41]. Importantly, regarding the most important nutrients, the digestate demonstrated a valuable composition that can compete with commercial fertilizers [55,56].

Similarly to the main nutrients regarding Ca and Mg, the introduction of co-substrates resulted in their increased contents in digestate, as compared to SS mono-digestion (R1). The content of both macro-elements in digested SS was related to the size of the WWTPs and adopted treatment technology. Generally, their contents did not exceed a level of 30 g/kg. Both metals are valuable and desirable components of fertilizers. They play an important role in physiological and biochemical functions of plants, e.g., growth and photosynthesis [57]. Additionally, previous studies indicated that the application of alkaline substances to contaminated soils may reduce the content of HMs [58,59].

In the case of Na, a lower content of this macro-element was obtained in R7 as compared to the control. In other cases, minor increases, not exceeding 10%, were found.

#### 3.2.2. Content of Micro-Elements

Despite the low concentrations of trace metals, they play a vital role in the AD process. Some of them, such as Ni, Fe and Mn, are crucial to the optimal growth of microbes and related methane production. On the other hand, micro-elements such as Zn, Cr, Pb and Cu can disturb enzymatic activities, leading even to process failure [60]. It should be pointed out that sole SS contains certain amounts of HMs. Therefore, co-substrates that indicate low levels of inhibitory compounds as well as dilution or/and absorption properties are of high interest [1]. In this regard, the use of food industrial by-products may be an interesting option.

The highest contents of all of the analyzed trace metals in feedstock were found in the control reactor that was supplied only with SS (Table 4). As compared to other SS obtained from the municipal WWTP, the applied material was characterized by high contents of Ni, Cu and Cr, and a relatively low level of Mn [28]. This finding was related to the characteristics of municipal wastewater influenced by the type of industry located in the settlement.

The introduction of co-substrates led to feedstock dilution, understood as reduced concentrations of analyzed trace elements in the co-digestion series. This mechanism has been proven by many researchers [1,2]. In almost all of the cases, the lowest metal concentration was observed in the three-component experiments (R6–R7). The major decreases varying between 54–60% were found in relation to Fe. The significant drops exceeding 40% were also observed in the three-component experiments in the case of Pb, Cd and Cu. With regard to Hg, a decrease exceeding 50% occurred in R8, which was supplied with the highest dose of PL and BSG.

The reduced content of HMs in the co-substrates was particularly beneficial in terms of AD, which is a highly complicated and sensitive process involving diverse groups of microorganisms [29]. HMs have been recognized as a substantial factor that influences the anaerobic activities of bacteria. Depending on their form and concentration, their effect on AD can be stimulatory, inhibitory, or even toxic [4].

In all of the series R1–R8 within AD, the concentrations of all analyzed trace metals increased as compared to those in the feedstock composition. This fact is associated with bioaccumulation and biosorption mechanisms [61]. The former is an active process that involves intracellular accumulation of metal, whereas in the latter—which is a passive process—metals are bound with the cellular surface. Besides the bioaccumulation and biosorption of heavy metals in the bodies of bacteria, heavy metals may also be adsorbed by an organic absorbent present in the sludge [62,63,64,65].

As shown in Figure 3, this effect was different in the individual series. The highest accumulation of trace metals in digestate was noticed in R6, which was accompanied by decreased biogas/methane production.

This fact may be related with the differences between microbial consortia in the reactors. Moreover, the effectiveness of both bioaccumulation and biosorption depends on the specific conditions in reactors, such as the pH or the presence of other competing ions [66]. However, further research should be conducted in this area.

As compared to the control (SS mono-digestion), in almost all of the AcoD series, enhanced contents of all analyzed metals in digestate were obtained. There was one exception concerning series R7, for which the concentration of all metals visibly decreased, similarly as in the case of some of its macro-nutrients. This observation might also confirm the synergetic effect that occurred in the presence of highest dose of PL and BSG.

Generally, the digestate concentrations of HMs were established at favorable levels for biogas production [67,68,69]. This did not apply to Fe and Zn. The former is used by methanogenic bacteria in the conversion of CO_2_ to CH_4_; it functions both as an electron acceptor and donor [70]. In the present study, higher values than those recommended for optimal biogas production were achieved, not exceeding a level of 8000 mg/L, which is considered inhibitory [4].

Zn is another crucial micro-element in the AD process; it plays an important role in hydrolysis and acidogenesis stages. Concentrations between 50 and 100 mg/L may disturb biogas production, while those above 400 mg/L promote VFA inhibition [70]. However, its toxic influence depends on many factors, e.g., properties of the applied substrate as well as adopted operating conditions, such as temperature, type of inoculum and the inoculum-to-substrate ratio [71]. In the present study, a relatively high level of Zn was observed in all of the reactors. This is related with an increased content of this metal in influent wastewater, which is typical under Polish conditions (galvanized pipes).

Importantly, the Ni and Cu contents were at favorable levels for effective biogas production. Ni is considered to be one of the most toxic among all HMs. Nonetheless, it is an essential trace element that is involved in both cellulase and methanogenic activities [68]. In turn, Cu is crucial to maintain the physiological activity of enzymes and microbes. Several studies indicated that low concentrations of Cu promote the degradation of VFA and enhance biogas production [72,73].

Regarding other HMs, low contents were achieved in all of the reactors. Previous studies indicated that concentrations above 32 mg/L generally resulted in lower biogas production [74].

In the case of Mn, its low content in digestate was observed. Thus far, few studies have considered the role of this metal within AD [75]. Some of them indicated that Mn is involved in the stabilization of methyltransferase as well as redox reactions [76,77]. In a study performed by Wang, a high content of Mn—varying between 5000–10,000 mg/kg—was found to promote the AD process [70].

Another aspect that should be considered corresponds to the potential environmental risks resulting from applying the residue as crop fertilizer. The levels of HMs in SS are strictly limited by the legal regulations applicable in a given country or union. As shown in Table 5, according to EU Directive 86/278/EEC [78], the permitted limits of Cu, Zn and Hg were exceeded, thus precluding their further agricultural use. However, the US regulations do not preclude their further agricultural use [79].

## 4. Conclusions

The implementation of an AcoD strategy requires an in-depth analysis of numerous factors. It should take into account technological, economic and environmental issues. The obtained results indicated that introduction of the highest doses of PL and BSG (R8) to municipal SS led to increased methane production as well as enhanced VRS, which were related to improved composition. On the contrary, in the three-component series supplied with OP and BSG (R6), reduced biogas and methane production as compared to the control was observed. Therein, the highest accumulation of most HMs within AD was also achieved, indicating the importance of selecting an adequate co-substrate. This fact also confirmed the relationship between the content of HMs and process performance. It should be pointed out that the presented results are preliminary, and should be further studied in semi-flow reactors and, finally, on a technical scale. Only such investigations will determine the general and long-term influences of co-substrate applications on process performance. Additionally, subsequent studies should focus on the application of additive/s that would eliminate the influence of the AD inhibitors, in particular in multi-component co-digestion with OP. Another pathway that should be investigated is the monitoring of microbial consortia occurring in particular reactors.

## Figures and Tables

**Table 1:** Composition of the co-substrates used in the experiment (average values and standard deviations are given).

Parameter	Unit	Orange Peels (OP)	Orange Pulp (PL)	Brewery Spent Grain (BSG)
Chemical oxygen demand (COD)	g/L	10.2 ± 0.3	11.8 ± 0.23	33.6± 0.68
Volatile fatty acids (VFA)	mg/L	611 ± 20.2	432.5 ± 15.2	608 ± 10.9
pH	-	4.35 ± 0.2	4.33 ± 0.1	5.01 ± 0.3
Alkalinity (ALK)	mg/L	27.1 ± 7.28	35.3 ± 6.08	242.0 ± 2.83
Total solids (TS)	g/kg	235.09 ± 0.50	155.18 ± 0.23	937.78 ± 0.32
Volatile solids (VS)	g/kg	224.41 ± 0.87	149.28 ± 0.47	878.21 ± 0.77
Macro-elements				
Total nitrogen (TN)	mg/L	92.1 ± 4.2	111.0 ± 6.1	739.4 ± 51.4
	g/kg	0.39 ± 0.05	0.72 ± 0.04	0.79 ± 0.01
Sodium (Na)	mg/L	57.0 ± 3.2	45.7 ± 2.3	82.9 ± 5.3
	g/kg	0.24 ± 0.05	0.29 ± 0.01	0.88 ± 0.03
Magnesium (Mg)	mg/L	171.7 ± 7.7	115.8 ± 8.7	259.8 ± 7.9
	g/kg	0.73 ± 0.02	0.75 ± 0.01	0.70 ± 0.035
Phosphorus (P)	mg/L	199.7 ± 4.9	369.8 ± 4.7	419.1 ± 49.7
	g/kg	0.85 ± 0.05	2.74 ± 0.02	4.47 ± 0.61
Potassium (K)	mg/L	2349 ± 36.1	1687 ± 10.1	1131 ± 8.7
	g/kg	9.99 ± 0.6	10.87 ± 1.3	10.21 ± 0.37
Calcium (Ca)	mg/L	1688 ± 13.1	887.8 ± 13.7	1248 ± 21.7
	g/kg	7.18 ± 1.3	5.72 ± 0.05	1.33 ± 0.09
Sulfur (S)	mg/L	203.3 ± 11.7	156.4 ± 9.8	207.8 ± 12.7
	g/kg	0.86 ± 0.05	1.01 ± 0.02	2.21 ± 0.03
Micro-elements				
Chromium (Cr)	mg/L	0.108 ± 0.05	0.114 ± 0.02	2.96 ± 0.05
	mg/kg	0.46 ± 0.01	0.73 ± 0.01	3.16 ± 0.01
Manganese (Mn)	mg/L	1.23 ± 0.06	0.525 ± 0.01	34.4 ± 0.3
	mg/kg	5.21 ± 0.01	3.39 ± 0.1	3.73 ± 0.3
Iron (Fe)	mg/L	39.75 ± 3.3	17.37 ± 1.2	144.5 ± 7.8
	mg/kg	169.10 ± 10.7	111.90 ± 10.7	154.1 ± 6.9
Nickel (Ni)	mg/L	0.21 ± 0.01	0.19 ± 0.05	0.57 ± 0.04
	mg/kg	0.91 ± 0.20	1.24 ± 0.07	0.61 ± 0.03
Copper (Cu)	mg/L	2.71 ± 0.01	1.43 ± 0.03	3.69 ± 0.03
	mg/kg	11.51 ± 0.05	9.18 ± 0.7	3.94 ± 0.03
Zinc (Zn)	mg/L	6.35 ± 0.04	6.17 ± 0.5	4.62 ± 5.1
	mg/kg	27.01 ± 1.2	39.77 ± 4.2	5.85 ± 4.9
Arsenic (As)	mg/L	0.003 ± 0.001	0.027 ± .0.001	0.050 ± 0.001
	mg/kg	0.013 ± 0.001	0.175 ± 0.002	0.053 ± 0.001
Cadmium (Cd)	mg/L	0.002 ± 0.001	0.002 ± 0.001	0.015 ± 0.001
	mg/kg	0.008 ± 0.003	0.011 ± 0.003	0.016 ± 0.001
Mercury (Hg)	mg/L	0.150 ± 0.004	0.16 ± 0.02	0.37 ± 0.02
	mg/kg	0.640 ± 0.04	1.02 ± 0.001	0.80 ± 0.03
Lead (Pb)	mg/L	0.049 ± 0.001	0.029 ± 0.001	0.026 ± 0.01
	mg/kg	0.21 ± 0.001	0.19 ± 0.01	0.28 ± 0.01

**Table 2:** Results of volatile solid removal (VRS) and stability parameters in the corresponding series (average values and standard deviations are given).

Reactor	Volatile Solid Removal (VRS) %	VFA/ALK -	pH -
R1	55.6 ± 4.7	0.052 ± 0.003	7.44 ± 0.01
R2	69.5 ± 4.1	0.058 ± 0.002	7.46 ± 0.01
R3	59.8 ± 4.0	0.048 ± 0.003	7.42 ± 0.02
R4	69.8 ± 4.6	0.056 ± 0.001	7.49 ± 0.03
R5	60.9 ± 3.2	0.048 ± 0.001	7.46 ± 0.01
R6	63.3 ± 1.8	0.058 ± 0.002	7.43 ± 0.01
R7	57.3 ± 1.1	0.066 ± 0.001	7.45 ± 0.02
R8	59.7 ± 1.3	0.062 ± 0.001	7.44 ± 0.02

**Table 3:** Contents of macro-elements in the experimental series (average values and standard deviations are given).

Reactor	Unit	N	Na	Mg	P	K	Ca	S
	Feedstock							
R1	mg/L	1074 ± 12.7	119.6 ± 5.1	146.5 ± 12.4	106.1 ± 8.8	182.9 ± 30.1	156.5 ± 7.7	317.9 ± 19.7
	g/kg	23.1 ± 8.1	1.7 ± 1.3	2.4 ± 0.1	3.7 ± 3.3	4.2 ± 3.4	2.5 ± 1.4	7.7 ± 3.5
R2	mg/L	1348 ± 14.5	120.3 ± 3.2	217.4 ± 7.2	331.2 ± 9.2	304.3 ± 27.5	186.7 ± 8.7	405.8 ± 9.2
	g/kg	28.3 ± 7.8	1.8 ± 0.05	4.8 ± 1.1	7.0 ± 1.5	6.4 ± 2.2	3.9 ± 1.4	7.5 ± 2.3
R3	mg/L	1335 ± 10.7	121.6 ± 7.6	254.9 ± 9.9	340.4 ± 4.6	342.9 ± 14.3	207.7 ± 10.4	404.3 ± 7.6
	g/kg	27.6 ± 6.9	1.9 ± 0.07	5.3 ± 1.5	7.4 ± 1.6	7.1 ± 2.3	4.3 ± 1.7	7.0 ± 2.4
R4	mg/L	1269 ± 8.7	123.7 ± 8.7	234.1 ± 10.4	335.3 ± 7.7	296.6 ± 20.1	200.9 ± 9.7	427.3 ± 7.9
	g/kg	26.5 ± 7.7	2.7 ± 0.07	4.9 ± 0.7	7.0 ± 2.1	6.2 ± 1.3	4.2 ± 1.1	8.9 ± 2.5
R5	mg/L	1338 ± 9.1	124.9 ± 10.3	183.4 ± 5.4	332.8 ± 6.9	301.0 ± 14.2	210.0 ± 10.4	423.2 ± 5.5
	g/kg	26.3 ± 8.1	2.3 ± 0.04	3.6 ± 0.3	4.6 ± 1.9	3.9 ± 1.4	4.1 ± 0.9	8.3 ± 1.7
R6	mg/L	1499 ± 13.7	126.2 ± 11.4	164.9 ± 7.3	198.4 ± 4.5	201.4 ± 9.8	185.6 ± 12.7	440.0 ± 4.5
	g/kg	27.8 ± 5.5	2.0 ± 0.03	3.1 ± 0.4	3.7 ± 1.2	6.0 ± 1.4	3.4 ± .08	8.3 ± 1.4
R7	mg/L	1507 ± 10.7	126.1 ± 7.9	247.8 ± 12.1	342.8 ± 12.7	357.5 ± 12.7	200.9 ± 14.8	441.0 ± 7.0
	g/kg	28.9 ± 6.1	2.4 ± 0.03	4.4 ± 0.4	9.9 ± 1.8	10.6 ± 1.7	4.5 ± 1.1	9.5 ± 1.7
R8	mg/L	1571 ± 10.3	129.7 ± 10.1	256.2 ± 10.3	364.2 ± 10.3	391.2 ± 13.4	215.6 ± 12.7	444.7 ± 6.9
	g/kg	30.0 ± 7.8	2.5 ± 0.02	4.3 ± 0.5	6.0 ± 1.9	6.1 ± 2.1	4.5 ± 1.0	10.7 ± 1.9
	Digestate							
R1	mg/L	1492 ± 8.8	261.4 ± 10.2	472.9 ± 10.1	519.2 ± 15.7	426.7 ± 20.1	323.8 ± 6.7	886.2 ± 10.8
	g/kg	66.7 ± 10.1	11.7 ± 1.2	21.1 ± 4.1	23.2 ± 3.2	19.1 ± 2.8	14.5 ± 1.4	39.6 ± 3.1
R2	mg/L	1769 ± 9.3	299.0 ± 7.8	558.6 ± 19.1	609.4 ± 17.2	508.6 ± 22.1	381.1 ± 12.7	1079.9 ± 10.4
	g/kg	85.0 ± 12.4	14.4 ± 3.1	26.8 ± 3.9	29.3 ± 1.2	24.4 ± 3.1	18.3 ± 1.3	51.9 ± 4.0
R3	mg/L	1663 ± 7.8	280.8 ± 14.5	543.2 ± 17.3	597.1 ± 14.2	486.8 ± 17.8	376.3 ± 14.7	1047.7 ± 12.1
	g/kg	74.8 ± 14.4	12.6 ± 2.7	24.4 ± 2.2	26.9 ± 4.2	21.9 ± 2.7	16.9 ± 1.8	47.2 ± 3.8
R4	mg/L	1653 ± 15.3	243.0 ± 16.7	499.2 ± 15.1	581.1 ± 17.3	428.6 ± 15.6	365.2 ± 13.6	999.2 ± 13.1
	g/kg	88.3 ± 13.2	13.0 ± 3.4	26.7 ± 4.6	31.0 ± 3.4	22.9 ± 2.9	19.5 ± 1.7	53.3 ± 4.7
R5	mg/L	1748 ± 12.8	265.6 ± 17.9	496.2 ± 14.3	572.1 ± 15.1	430.7 ± 17.7	371.7 ± 14.9	965.2 ± 14.2
	g/kg	77.2 ± 10.4	11.7 ± 2.7	21.9 ± 5.2	25.3 ± 4.1	19.0 ± 2.5	16.4 ± 2.0	42.7 ± 5.0
R6	mg/L	1169 ± 8.8	280.4 ± 10.3	505.1 ± 12.4	575.1 ± 15.1	466.0 ± 16.4	360.9 ± 15.7	969.3 ± 14.3
	g/kg	53.1 ± 7.7	12.7 ± 3.1	22.9 ± 5.1	26.1 ± 3.4	21.2 ± 1.8	16.4 ± 1.7	44.0 ± 3.3
R7	mg/L	1330 ± 10.1	242.9 ± 11.3	495.0 ± 14.4	591.0 ± 22.5	453.9 ± 13.4	362.2 ± 12.1	954.8 ± 9.8
	g/kg	49.4 ± 8.9	9.0 ± 1.5	18.4 ± 4.3	26.0 ± 2.8	24.4 ± 1.9	13.5 ± 1.3	35.5 ± 3.4
R8	mg/L	1247 ± 10.5	269.2 ± 7.9	507.1 ± 20.1	551.0 ± 24.0	459.3 ± 17.5	343.9 ± 17.1	943.4 ± 8.9
	g/kg	63.1 ± 7.6	13.6 ± 3.7	25.7 ± 7.8	27.9 ± 2.4	23.2 ± 2.1	17.4 ± 1.4	47.7 ± 4.1

**Table 4:** Contents of micro-elements in the experimental series (average values and standard deviations are given).

Reactor	Unit	Cr	Mn	Fe	Ni	Cu	Zn	As	Cd	Hg	Pb
Feedstock											
R1	mg/L	3.31 ± 0.1	2.39 ± 0.01	116.9 ± 12.2	1.10 ± 0.01	26.21 ± 2.3	70.31 ± 5.6	0.24 ± 0.01	0.15 ± 0.01	0.58 ± 0.05	0.78 ± 0.20
	mg/kg	71.12 ± 5.4	51.42 ± 2.2	2512 ± 60.1	23.58 ± 1.3	562.9 ± 10.3	1510 ± 37.8	5.21 ± 0.05	3.14 ± 0.7	12.54 ± 1.2	16.82 ± 2.1
R2	mg/L	2.29 ± 0.05	1.67 ± 0.04	62.25 ± 7.8	0.85 ± 0.5	20.96 ± 1.3	64.68 ± 4.3	0.20 ± 0.03	0.10 ± 0.01	0.35 ± 0.07	0.57 ± 0.01
	mg/kg	48.11 ± 3.6	35.08 ± 2.1	1307 ± 10.7	17.79 ± 2.2	440.2 ± 12.4	1358 ± 27.1	4.14 ± 0.9	2.08 ± 0.05	7.25 ± 0.7	11.91 ± 2.1
R3	mg/L	2.60 ± 0.03	1.87 ± 0.01	69.29 ± 5.5	0.95 ± 0.04	22.60 ± 5.6	61.99 ± 3.6	0.21 ± 0.02	0.13 ± 0.02	0.55 ± 0.04	0.65 ± 0.05
	mg/kg	53.80 ± 2.2	38.70 ± 1.4	1432 ± 15.7	19.72 ± 5.4	467.1 ± 14.3	1281 ± 26.3	4.29 ± 0.7	2.63 ± 0.07	11.40 ± 2.1	13.52 ± 2.5
R4	mg/L	2.39 ± 0.01	1.71 ± 0.02	66.32 ± 8.1	0.89 ± 0.05	20.83 ± 3.6	57.79 ± 4.7	0.20 ± 0.01	0.11 ± 0.03	0.36 ± 0.02	0.57 ± 0.09
	mg/kg	50.00 ± 3.7	35.71 ± 1.7	1385 ± 25.1	18.52 ± 3.4	435.1 ± 14.7	1207 ± 45.1	4.11 ± 0.5	2.24 ± 0.04	7.61 ± 0.7	11.96 ± 2.1
R5	mg/L	2.25 ± 0.02	1.30 ± 0.02	62.82 ± 5.4	0.78 ± 0.05	18.01 ± 3.6	60.81 ± 3.7	0.16 ± 0.01	0.12 ± 0.01	0.41 ± 0.03	0.69 ± 0.07
	mg/kg	44.19 ± 3.1	25.50 ± 1.5	1234 ± 14.7	15.38 ± 2.5	353.6 ± 25.6	1194 ±44.3	3.07 ± 0.75	2.35 ± 0.03	8.06 ± 1.3	13.50 ± 2.7
R6	mg/L	1.95 ± 0.01	1.06 ± 0.02	54.97 ± 7.7	0.72 ± 0.08	15.58 ± 3.6	51.59 ± 3.7	0.13 ± 0.02	0.09 ± 0.01	0.51 ± 0.05	0.53 ± 0.06
	mg/kg	36.24 ± 1.3	19.70 ± 1.2	1020 ± 25.6	13.28 ± 3.6	289.2 ± 10.1	957.5 ± 17.9	2.44 ± 0.07	1.74 ± 0.04	9.50 ± 2.1	9.92 ± 0.5
R7	mg/L	2.97 ± 0.03	1.61 ± 0.02	57.99 ± 4.7	0.76 ± 0.05	18.46 ± 3.4	50.56 ± 3.7	0.17 ± 0.01	0.09 ± 0.02	0.41 ± 0.07	0.50 ± 0.04
	mg/kg	56.88 ± 5.1	30.93 ± 4.7	1112 ±36.7	14.59 ± 2.4	354.0 ± 9.7	969.7 ± 22.4	3.31 ± 0.03	1.77 ± 0.07	7.92 ± 1.4	9.56 ± 1.1
R8	mg/L	2.45 ± 0.01	1.65 ± 0.2	60.93 ± 5.4	0.80 ± 0.03	19.61 ± 3.6	52.39 ± 4.1	0.18 ± 0.01	0.10 ± 0.02	0.31 ± 0.04	0.52 ± 0.03
	mg/kg	46.71 ± 3.2	31.46 ± 3.2	1163 ± 19.3	15.18 ± 1.6	374.5 ± 12.6	1000 ± 40.2	3.41 ± 0.02	1.89 ± 0.04	5.95 ± 0.75	9.90 ± 1.7
Digestate											
R1	mg/L	3.50 ± 0.5	2.96 ± 0.01	108.2 ± 7.9	1.38 ± 0.02	33.48 ± 2.7	96.25 ± 8.8	0.32 ± 0.02	0.18 ± 0.02	0.52 ± 0.06	0.95 ± 0.05
	mg/kg	156.58 ± 10.4	132.51 ± 21.3	4836 ± 44.1	61.67 ± 3.2	1496.7 ± 36.1	4303 ±45.7	14.11 ± 3.2	8.09 ± 0.4	23.07 ± 1.3	42.38 ± 1.3
R2	mg/L	4.05 ± 0.07	3.51 ± 0.2	125.0 ± 10.7	1.63 ± 0.04	39.90 ± 5.1	103.6 ± 7.8	0.37 ± 0.04	0.22 ± 0.02	0.71 ± 0.03	1.13 ± 0.01
	mg/kg	194.56 ± 11.7	168.58 ± 17.2	6008 ± 75.3	78.09 ± 3.4	1917 ± 45.2	4980 ± 39.7	17.93 ± 2.2	10.76 ± 1.1	34.29 ± 2.1	54.10 ± 2.3
R3	mg/L	4.07 ± 0.09	3.45 ± 0.05	124.7 ± 14.3	1.58 ± 0.05	39.02 ± 5.4	101.8 ± 10.2	0.38 ± 0.04	0.21 ± 0.03	0.64 ± 0.03	1.11 ± 0.05
	mg/kg	183.10 ± 9.7	155.26 ± 10.2	5612 ± 36.7	71.30 ± 4.1	1756 ± 47.2	4581 ± 56.3	17.10 ± 2.1	9.66 ± 1.1	28.95 ± 2.8	49.75 ± 2.2
R4	mg/L	4.07 ± 0.07	3.38 ± 0.07	125.2 ± 7.18	1.52 ± 0.06	38.57 ± 4.1	101.5 ± 10.7	0.35 ± 0.07	0.20 ± 0.01	0.70 ± 0.03	1.20 ± 0.03
	mg/kg	217.47 ± 14.7	180.53 ± 26.3	6685. ±32.2	81.38 ± 6.7	2059 ± 10.2	5417 ± 53.3	18.83 ± 2.3	10.79 ± 2.1	37.16 ± 2.2	63.83 ± 2.1
R5	mg/L	3.83 ± 0.7	3.20 ± 0.07	124.9 ± 7.2	1.51 ± 0.04	40.10 ± 5.1	125.1 ± 3.8	0.33 ± 0.08	0.21 ± 0.02	0.63 ± 0.07	1.13 ± 0.05
	mg/kg	169.23 ± 14.5	141.47 ± 12.3	5518 ± 44.3	66.88 ± 4.2	1772 ± 35.1	5530 ± 40.5	14.65 ± 2.3	9.42 ± 1.2	27.62 ± 1.9	50.10 ± 2.3
R6	mg/L	3.78 ± 0.8	3.33 ± 0.05	125.1 ± 12.5	1.51 ± 0.04	39.32 ± 4.1	109.7 ± 7.7	0.35 ± 0.07	0.20 ± 0.02	0.64 ± 0.03	1.06 ± 0.07
	mg/kg	171.40 ± 16.7	151.00 ± 14.1	5677 ± 45	68.75 ± 3.8	1785 ± 38.7	4980 ± 55.1	15.90 ± 3.2	8.98 ± 0.75	29.16 ± 2.2	48.17 ± 3.5
R7	mg/L	3.86 ± 0.6	3.36 ± 0.04	124.5 ± 10.3	1.53 ± 0.05	37.30 ± 2.1	99.24 ± 7.2	0.33 ± 0.01	0.20 ± 0.01	0.53 ± 0.03	1.02 ± 0.02
	mg/kg	143.65 ± 20.1	124.78 ± 10.2	4629 ± 51.3	56.90 ± 4.7	1387 ± 24.1	3689 ± 41.3	12.15 ± 2.1	7.57 ± 0.65	19.62 ± 0.8	39.90 ± 1.1
R8	mg/L	3.56 ± 0.04	3.10 ± 0.07	112.3 ± 8.1	1.51 ± 0.04	35.48 ± 3.7	97.43 ± 7.7	0.33 ± 0.06	0.17 ± 0.02	0.55 ± 0.02	0.97 ± 0.05
	mg/kg	180.09 ± 10.3	157.05 ± 12.1	5683 ± 31.2	76.63 ± 6.4	1796 ± 45.2	4931 ± 37.5	16.79 ± 2.2	8.81 ± 0.9	23.74 ± 1.5	18.60 ± 1.2

**Table 5:** The allowable limits of HMs [mg/kg] in the EU and US [78,79].

Heavy Metal	EU Regulation	US Regulation	This Study
Cd	20–40	85	7.6–10.8
Cu	1000–1750	4300	1387–2059
Ni	300–400	420	61.7–81.4
Pb	750–1200	840	18.6–63.8
Zn	2500–4500	7500	3689–5530
Hg	16–25	57	19.6–37.2

## Data Availability

Not applicable.

## References

[1] Mata-Álvarez J., Dosta J., Romero-Guiza M.S., Fonoll X., Peces M., Astals S. (2014). A critical review on anaerobic co-digestion achievements between 2010 and 2013. Renew. Sustain. Energ. Rev..

[2] Rabii A., Aldin S., Dahman Y., Elbeshbishy E. (2019). A Review on Anaerobic Co-Digestion with a Focus on the Microbial Populations and the Effect of Multi-Stage Digester Configuration. Energies.

[3] Chen Y., Cheng J.J., Creamer K.S. (2008). Inhibition of anaerobic digestion process: A review. Bioresour. Technol..

[4] Lee J., Park K.Y., Cho J., Kwon E.E., Kim J.Y. (2018). Anaerobic digestion as an alternative disposal for phytoremediated biomass from heavy metal contaminated sites. Environ. Pollut..

[5] Oleszkiewicz J.A., Sharma V.K. (1990). Stimulation and inhibition of anaerobic processes by heavy metals—A review. Biol. Wastes.

[6] Jin K., Ran Y., Alengebawy A., Yang G., Jia S., Ai P. (2020). Agro-environmental sustainability of using digestate fertilizer for solanaceous and leafy vegetables cultivation: Insights on fertilizer efficiency and risk assessment. J. Environ..

[7] Ai P., Jin K., Alengebawy A., Elsayed M., Ran Y. (2020). Effect of application of different biogas fertilizer on eggplant production: Analysis of fertilizer value and risk assessment. Environ. Technol. Innovat..

[8] Zhang Q., Zou D., Zeng X., Li L., Xiao Z. (2020). Effect of the direct use of biomass in agricultural soil on heavy metals—Activation or immobilization?. Environ. Pollut..

[9] Suciu N.A., Lamastra L., Trevisan M. (2015). PAHs content of sewage sludge in Europe and its use as soil fertilizer. Waste Manag..

[10] Homepage—FoodDrinkEurope. https://www.fooddrinkeurope.eu/.

[11] Rezzadori K., Benedetti S., Amante E.R. (2012). Proposals for the residues recovery: Orange waste as raw material for new products. Food Bioprod..

[12] Bachmann S.A., Calvete T., Féris L.A. (2021). Potential applications of brewery spent grain: Critical an overview. J. Environ. Chem. Eng..

[13] Barros R.S., Contreras M.P., Morris F.R., Chamorro M.V., Arrieta A.R. (2023). Evaluation of the methanogenic potential of anaerobic digestion of agro-industrial wastes. Heliyon.

[14] Bedoić R., Spehar A.B., Puljko J., Čuček L., Ćosić B., Pukšec T., Duić N. (2020). Opportunities and challenges: Experimental and kinetic analysis of anaerobic co-digestion of food waste and rendering industry streams for biogas production. Renew. Sustain. Energy Rev..

[15] Carlini M., Monarca D., Castellucci S., Mennuni A., Casini L., Selli S. (2021). Beer spent grains biomass for biogas production: Characterization and anaerobic digestion-oriented pre-treatments. Energy Rep..

[16] Martínez E.J., Rosas J.G., Sotres A., Morán A., Cara J., Sánchez M.E., Gómez X. (2018). Codigestion of sludge and citrus peel wastes: Evaluating the effect of biochar addition on microbial communities. Biochem. Eng. J..

[17] Bougrier C., Dognin D., Laroche C., Gonzalez V., Benali-Raclot D., Cacho Rivero J.A. (2018). Anaerobic digestion of Brewery Spent Grains: Trace elements addition requirement. Bioresour. Technol..

[18] American Public Health Association (APHA) (2012). Standard Methods for the Examination of Water and Wastewater.

[19] Bernard M., Polit M., Hadj-Sadok Z., Pengov M., Dochain D., Estaben M., Labat P. (2001). Advanced monitoring and control of anaerobic wastewater treatment plants: Software sensors and controllers for an anaerobic digester. Water Sci. Technol..

[20] Issah A.A., Kabera T. (2021). Impact of volatile fatty acids to alkalinity ratio and volatile solids on biogas production under thermophilic conditions. Waste Manag. Res. J. Sustain. Circ. Econ..

[21] Ahring B.K., Angelidaki I. Monitoring and controlling the biogas process. Proceedings of the 8th International Conference on Anaerobic Digestion.

[22] Szaja A., Montusiewicz A. (2019). Enhancing the co-digestion efficiency of sewage sludge and cheese whey using brewery spent grain as an additional substrate. Bioresour. Technol..

[23] Szaja A., Montusiewicz A., Pasieczna-Patkowska S., Lebiocka M. (2022). Technological and Energetic Aspects of Multi-Component Co-Digestion of the Beverage Industry Wastes and Municipal Sewage Sludge. Energies.

[24] Ruiz B., Flotats X. (2016). Effect of limonene on batch anaerobic digestion of citrus peel waste. Biochem. Eng. J..

[25] Negro V., Mancini G., Ruggeri B., Fino D. (2016). Citrus waste as feedstock for bio-based products recovery: Review on limonene case study and energy valorization. Bioresour. Technol..

[26] Jung S., Kim M., Lee J., Shin J., Shin S.G., Lee J. (2022). Effect of magnetite supplementation on mesophilic anaerobic digestion of phenol and benzoate: Methane production rate and microbial communities. Bioresour. Technol..

[27] Martín M.A., Fernández R., Gutiérrez M.C., Siles J.A. (2018). Thermophilic anaerobic digestion of pre-treated orange peel: Modelling of methane production. Process. Saf. Environ. Prot..

[28] Seleiman M.F., Santanen A., Mäkelä P.S. (2020). Recycling sludge on cropland as fertilizer—Advantages and risks. Resour. Conserv. Recycl..

[29] Hagos K., Zong J., Li D., Liu C., Lu X. (2017). Anaerobic co-digestion process for biogas production: Progress, challenges and perspectives. Renew. Sustain. Energy Rev..

[30] Vintiloiu A., Lemmer A., Oechsner H., Jungbluth T. (2012). Mineral substances and macronutrients in the anaerobic conversion of biomass: An impact evaluation. Eng. Life Sci..

[31] Banks C.J., Heaven S., Wellinger A., Murphy J., Baxter D. (2013). Optimisation of biogas yields from anaerobic digestion by feedstock type. The Biogas Handbook.

[32] Wang X., Yang G., Feng Y., Ren G., Han X. (2012). Optimizing feeding composition and carbon-nitrogen ratios for improved methane yield during anaerobic co-digestion of dairy, chicken manure and wheat straw. Bioresour. Technol..

[33] Fermoso F.G., van Hullebusch E.D., Guibaud G., Collins G., Svensson B.H., Carliell-Marquet C., Vink J.P., Esposito G., Frunzo L. (2015). Fate of Trace Metals in Anaerobic Digestion. Adv. Biochem. Eng. Biotechnol..

[34] Hussain A., Kumar P., Mehrotra I. (2015). Nitrogen and phosphorus requirement in anaerobic process: A review. Environ. Eng. Manag. J..

[35] Fernandez N., Forster C.F. (1994). The anaerobic digestion of simulated coffee waste using thermophilic and mesophilic upflow filters. Process. Saf. Environ. Protect..

[36] Lo H.M., Chiu H.Y., Lo S.W., Lo F.C. (2012). Effects of different SRT on anaerobic digestion of MSW dosed with various MSWI ashes. Bioresour. Technol..

[37] Tan L., Qu Y., Zhou J., Ma F., Li A. (2009). Dynamics of microbial community for X-3B wastewater decolorization coping with high-salt and metal ions conditions. Bioresour. Technol..

[38] Thiele J.H., Wu W.-M., Jain M.K., Zeikus J.G. (1990). Ecoengineering high rate anaerobic digestion systems: Analysis of improved syntrophic biomethanation catalysts. Biotechnol. Bioeng..

[39] Huang J., Pinder K.L. (1995). Effects of calcium on development of anaerobic acidogenic biofilms. Biotechnol. Bioeng..

[40] Yuan Z., Yang H., Zhi X., Shen J. (2009). Increased performance of continous stirred tank reactor with calcium supplementation. Int. J. Hydrog. Energy.

[41] Dewil R., Baeyens J., Roels J., Steene B.V. (2009). Evolution of the Total Sulphur Content in Full-Scale Wastewater Sludge Treatment. Environ. Eng. Sci..

[42] Romero-Güiza M.S., Vila J., Mata-Álvarez J., Chimenos J.M., Astals S. (2016). The role of additives on anaerobic digestion: A review. Renew. Sustain. Energy Rev..

[43] Mussatto S.I. (2014). Brewer’s spent grain: A valuable feedstock for industrial applications. J. Sci. Food Agric..

[44] Andreoli C.V., Pegorini E.S., Fernandes F., Santos H.F., Von Sperling M., Andreoli C.V., Fernandes F. (2007). Land application of sewage sludge. Sludge Treatment and Disposal.

[45] European Commission (2020). Communication From the Commission to the European Parliament, the Council, the European Economic and Social Committee and the Committee of the Regions: A New Circular Economy Action Plan for a Cleaner and More Competitive Europe.

[46] Urbaniak M., Wyrwicka A., Tołoczko W., Serwecińska L., Zieliński M. (2017). The effect of sewage sludge application on soil properties and willow (Salix sp.) cultivation. Sci. Total Environ..

[47] Koszel M., Lorencowicz E. (2015). Agricultural use of biogas digestate as a replacement fertilizers. Agric. Agric. Sci. Procedia.

[48] Kazimierowicz J. (2014). Organic waste used in agricultural biogas plants. J. Ecol. Eng..

[49] Nowak M., Kacprzak M., Grobelak A. (2010). Sewage sludge as a substitute for soil in the process of remediation and reclamation of sites contaminated with heavy metals. Eng. Environ. Prot..

[50] Stefaniuk M., Oleszczuk P. (2016). Addition of biochar to sewage sludge decreases freely dissolved PAHs content and toxicity of sewage sludge-amended soil. Environ. Pollut..

[51] Zielińska A., Oleszczuk P. (2015). Evaluation of sewage sludge and slow pyrolyzed sewage sludge-derived biochar for adsorption of phenanthrene and pyrene. Bioresour. Technol..

[52] Johnson R., Vishwakarma K., Hossen M.S., Kumar V., Shackira A.M., Puthur J.T., Abdi G., Sarraf M., Hasanuzzaman M. (2022). Potassium in plants: Growth regulation, signaling, and environmental stress tolerance. Plant Physiol. Biochem..

[53] Anjum N.A., Umar S., Singh S., Nazar R., Khan N.A., Khan N.A., Singh S., Umar S. (2008). Sulfur assimilation and cadmium tolerance in plants. Sulfur Assimilation and Abiotic Stress in Plants.

[54] Zhang D., Du G., Chen D., Shi G., Rao W., Li X., Jiang Y., Liu S., Wang D. (2019). Effect of elemental sulfur and gypsum application on the bioavailability and redistribution of cadmium during rice growth. Sci. Total Environ..

[55] Han F., Yun S., Zhang C., Xu H., Wang Z. (2019). Steel slag as accelerant in anaerobic digestion for nonhazardous treatment and digestate fertilizer utilization. Bioresour. Technol..

[56] Zhang C., Yun S., Li X., Wang Z., Xu H., Du T. (2018). Low-cost composited accelerants for anaerobic digestion of dairy manure: Focusing on methane yield, digestate utilization and energy evaluation. Bioresour. Technol..

[57] Djabou A.S.M., Qin Y., Thaddee B., Figueiredo P.G., Feifei A., Carvalho L.J.C.B., Omokolo D.N., Li K., Niemenak N., Chen S. (2018). Effects of Calcium and Magnesium Fertilization on Antioxidant Activities during Cassava Postharvest Physiological Deterioration. Crop Sci..

[58] Lominchar M.A., Santos A., de Miguel E., Romero A. (2018). Remediation of aged diesel contaminated soil by alkaline activated persulfate. Sci. Total Environ..

[59] Zeng X., Zou D., Wang A., Zhou Y., Liu Y., Li Z., Liu F., Wang H., Zeng Q., Xiao Z. (2020). Remediation of cadmium-contaminated soils using Brassica napus: Effect of nitrogen fertilizers. J. Environ. Manag..

[60] Alrawashdeh K.A.B. (2022). Anaerobic Co-digestion efficiency under the stress exerted by different heavy metals concentration: An energy nexus analysis. Energy Nexus.

[61] Chojnacka K. (2010). Biosorption and bioaccumulation—The prospects for practical applications. Environ. Int..

[62] Shamsollahi H.R., Alimohammadi M., Momeni S., Naddafi K., Nabizadeh R., Khorasgani F.C., Yousefi M. (2018). Assessment of the Health Risk Induced by Accumulated Heavy Metals from Anaerobic Digestion of Biological Sludge of the Lettuce. Biol. Trace Elem. Res..

[63] Inyang M., Gao B., Yao Y., Xue Y., Zimmerman A.R., Pullammanappallil P., Cao X. (2012). Removal of heavy metals from aqueous solution by biochars derived from anaerobically digested biomass. Bioresour. Technol..

[64] Alrawashdeh K.A., Gul E., Yang Q., Yang H., Bartocci P., Fantozzi F. (2020). Effect of Heavy Metals in the Performance of Anaerobic Digestion of Olive Mill Waste. Processes.

[65] Abdel-Shafy H.I., Mansour M.S. (2014). Mansour Biogas production as affected by heavy metals in the anaerobic digestion of sludge. Egypt. J. Pet..

[66] Naja G., Murphy V., Volesky B. (2010). Biosorption, Metals. Wiley Encyclopedia of Industrial Biotechnology.

[67] Guo Q., Majeed S., Xu R., Zhang K., Kakade A., Khan A., Hafeez F.Y., Mao C., Liu P., Li X. (2019). Heavy metals interact with the microbial community and affect biogas production in anaerobic digestion: A review. J. Environ. Manag..

[68] Tian Y., Zhang H., Chai Y., Wang L., Mi X., Zhang L., Ware M.A. (2017). Biogas properties and enzymatic analysis during anaerobic fermentation of Phragmites australis straw and cow dung: Influence of nickel chloride supplement. Biodegradation.

[69] Wang S., Wang J., Li J., Hou Y., Shi L., Lian C., Shen Z., Chen Y. (2021). Evaluation of biogas production potential of trace element-contaminated plants via anaerobic digestion. Ecotoxicol. Environ. Saf..

[70] Thanh P.M., Ketheesan B., Yan Z., Stuckey D. (2016). Trace metal speciation and bioavailability in anaerobic digestion: A review. Biotechnol. Adv..

[71] Huang Z., Niu Q., Nie W., Li X., Yang C. (2022). Effects of heavy metals and antibiotics on performances and mechanisms of anaerobic digestion. Bioresour. Technol..

[72] Facchin V., Cavinato C., Fatone F., Pavan P., Cecchi F., Bolzonella D. (2013). Effect of trace element supplementation on the mesophilic anaerobic digestion of foodwaste in batch trials: The influence of inoculum origin. Biochem. Eng. J..

[73] Karlsson A., Einarsson P., Schnürer A., Sundberg C., Ejlertsson J., Svensson B.H. (2012). Impact of trace element addition on degradation efficiency of volatile fatty acids, oleic acid and phenyl acetate and on microbial populations in a biogas digester. J. Biosci. Bioeng..

[74] Altaş L. (2009). Inhibitory effect of heavy metals on methane-producing anaerobic granular sludge. J. Hazard. Mater..

[75] Cai Y., Zheng Z., Zhao Y., Zhang Y., Guo S., Cui Z., Wang X. (2018). Effects of molybdenum, selenium and manganese supplementation on the performance of anaerobic digestion and the characteristics of bacterial community in acidogenic stage. Bioresour. Technol..

[76] Perry R.D., Silver S. (1982). Cadmium and manganese transport in Staphylococcus aureas membrane vesicles. J. Bacteriol..

[77] Fisher F., Bauxbaum L., Toth K., Eisenstadt E., Silver S. (1973). Regulation of manganese accumulation and exchange in *Bacillus subtilis* W23. J. Bacteriol..

[78] Council Directive 86/278/EEC of 12 June 1986 on the Protection of the Environment, and in Particular of the Soil, when Sewage Sludge is Used in Agriculture. EU Directive 86/278/EEC. http://data.europa.eu/eli/dir/1986/278/2022-01-01.

[79] US EPA (1993). 40FR Part 503—Standards for Use and Disposal of Sewage Sludge: Final Rules. Fed. Regist..

